# α-Costic anhydride

**DOI:** 10.1107/S1600536810005064

**Published:** 2010-02-13

**Authors:** Mohamed Tebbaa, Mohamed Akssira, Ahmed Elhakmaoui, Lahcen El Ammari, Ahmed Benharref, Moha Berraho

**Affiliations:** a Laboratoire de Chimie Bioorganique et Analytique, Faculté des Sciences et Techniques, 20800 Mohammedia, Morocco; b Laboratoire de Chimie du Solide Appliquée, Faculté des Sciences, Avenue Ibn Battouta BP 1014 Rabat, Morocco; c Laboratoire de Chimie des Substances Naturelles, Faculté des Sciences Semlalia, BP 2390 Bd My Abdellah, 40000 Marrakech, Morocco

## Abstract

The title compound [systematic name: 2-(4a,8-dimethyl-1,2,3,4,4a,5,6,8a-octa­hydro­naphthalen-2-yl)acrylic acid anhydride], C_30_H_42_O_3_, is a new isocostic anhydride which was synthesized from the aerial part of *Inula Viscosa­* (L) Aiton [or *Dittrichia Viscosa­* (L) Greuter]. The mol­ecule adopts an essentially linear shape with two terminal fused-rings bridged by the anhydride group. The external rings have the same conformation (half-chair) while each of the two inner rings has an almost ideal chair conformation. In the crystal, inter­molecular C—H⋯O inter­actions link the mol­ecules into a two-dimensional array in the *bc* plane.

## Related literature

For background to the medicinal inter­est in *Inula Viscosa­* (L) Aiton [or *Dittrichia Viscosa­* (L) Greuter], see: Shtacher & Kasshman (1970 ▶); Bohlman & Gupta (1982 ▶); Azoulay *et al.* (1986 ▶); Bohlmann *et al.* (1977 ▶); Ceccherelli *et al.* (1988 ▶); Grande *et al.* (1985 ▶); Chiappini *et al.* (1982 ▶). For background to the phytochemical study of Moroccan plants, see: Tebaa *et al.* (2009 ▶); Zeroual *et al.* (2007 ▶). For conformational analysis, see: Cremer & Pople (1975 ▶).
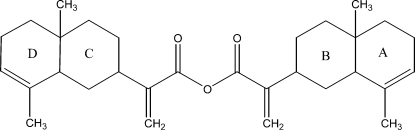


## Experimental

### 

#### Crystal data

C_30_H_42_O_3_
*M* *_r_* = 450.64
Monoclinic, 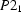

*a* = 6.6699 (2) Å
*b* = 6.6335 (2) Å
*c* = 30.2948 (8) Å
β = 92.799 (1)°
*V* = 1338.79 (7) Å^3^
*Z* = 2
Mo *K*α radiation
μ = 0.07 mm^−1^
*T* = 298 K
0.28 × 0.17 × 0.12 mm

#### Data collection

Bruker X8 APEX CCD area-detector diffractometer
15300 measured reflections
2914 independent reflections
2604 reflections with *I* > 2σ(*I*)
*R*_int_ = 0.030

#### Refinement

*R*[*F* ^2^ > 2σ(*F* ^2^)] = 0.043
*wR*(*F* ^2^) = 0.109
*S* = 1.05
2914 reflections
304 parameters
1 restraint
H-atom parameters constrained
Δρ_max_ = 0.17 e Å^−3^
Δρ_min_ = −0.12 e Å^−3^

### 

Data collection: *APEX2* (Bruker, 2009 ▶); cell refinement: *SAINT-Plus* (Bruker, 2009 ▶); data reduction: *SAINT-Plus*; program(s) used to solve structure: *SHELXS97* (Sheldrick, 2008 ▶); program(s) used to refine structure: *SHELXL97* (Sheldrick, 2008 ▶); molecular graphics: *ORTEP-3 for Windows* (Farrugia, 1997 ▶) and *DIAMOND* (Brandenburg, 2006 ▶); software used to prepare material for publication: *WinGX* publication routines (Farrugia, 1999 ▶).

## Supplementary Material

Crystal structure: contains datablocks I, global. DOI: 10.1107/S1600536810005064/tk2617sup1.cif

Structure factors: contains datablocks I. DOI: 10.1107/S1600536810005064/tk2617Isup2.hkl

Additional supplementary materials:  crystallographic information; 3D view; checkCIF report

## Figures and Tables

**Table 1:** Hydrogen-bond geometry (Å, °)

*D*—H⋯*A*	*D*—H	H⋯*A*	*D*⋯*A*	*D*—H⋯*A*
C14—H14*B*⋯O2^i^	0.93	2.47	3.378 (4)	166
C18—H18*B*⋯O3^ii^	0.93	2.51	3.419 (3)	168

Symmetry codes: (i) 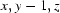; (ii) 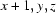.

**Fig. 1.**
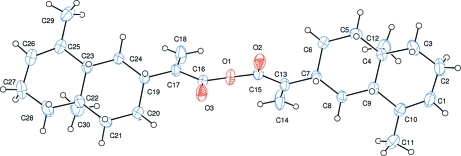
: Molecular structure of (I) showing the atom-labelling scheme. Displacement ellipsoids are drawn at the 30% probability level. H atoms are represented as small spheres of arbitrary radii.

**Fig. 2.**
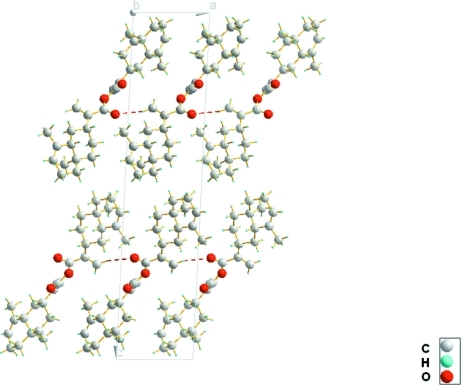
: A partial packing digram of (I), showing intermolecular C–H···O contacts (dashed lines). [Symmetry code: (i) x, -1+y, z; (ii) -1+x, y, z]

## supplementary crystallographic information

### Comment

Inula Viscosa (L) Aiton or Dittrichia Viscosa (L) Greuter is widespread in Mediterranean area and extends to the Atlantic cost of Morocco. It is a well known medicinal plant (Shtacher & Kasshman, 1970 ; Bohlman & Gupta, 1982) and has some pharmacological activities (Azoulay *et al.*, 1986). This plant has been the subject of chemical investigation in terms of isolating sesquiterpene lactones (Bohlmann *et al.*, 1977), sesquiterpene acids (Ceccherelli *et al.*,1988) and flavonoids (Grande *et al.*, 1985; Chiappini *et al.*, 1982).

The work of our research group has focused upon the phytochemical study of Moroccan plants (Tebaa *et al.*, 2009 ; Zeroual *et al.*, 2007) with the aim to discover new compounds which could be used as precursors or intermediates for the synthesis of novel molecules. In this way, we have investigated Inula Viscosa (L) which is rich in sesquiterpene derivatives. The title compound C_30_H_42_O_3_, (I), was obtained through a chemical modification of 2-(4a,8-dimethyl-1,2,3,4,4a,,5,6,8a-octahdro-naphtaen-2-yl)- acrylic acid,which was isolated in high yield from Inula viscosa (L). The dimerisation of the above compound was obtained by the treatment of iscostic acid by the ethyl chloroformate in the presence of triethylamine.

The molecular structure of (I), Fig. 1, shows each of the external rings (labelled A and D in the Scheme) to adopt a half-chair conformation, as indicated by the total puckering amplitude QT = 0.504 (3) Å and spherical polar angle θ = 131.4 (3) ° with φ = 286.2 (4) ° for ring (A), and QT = 0.504 (2) Å and θ =131.1 (3) ° with φ = 168.5 (4) ° for ring (D). By contrast, the inners rings (B and C) have a chair conformation with QT = 0.57 (2) Å, θ =1.3 (2) °,φ = 67 (5) ° for ring (B), and QT = 0.57 (2) Å, θ =2.0 (2) °, φ = 168.5 (4) ° for ring (C) (Cremer & Pople, 1975). In the crystal structure, there are two intermolecular C–H···O contacts, involving the carbonyl-O2 and -O3 atoms (Fig. 2; Table 1), which cooperate to form a 2-D array in the *bc* plane.

### Experimental

A solution containing equimolar quantities of isocostic acid (500 mg) and triethyl amine (0.5 mL) was stirred at 263 K for 10 mins. To this was added a 0.5 equivalent of ethyl chloroformate (0.3 mL) and the reaction mixture was stirred for 1 h. The residue obtained was purified on a silica gel column using hexane-ethyl acetate (99:1) as an eluent which yielded compound (I) in 70% yield.

### Refinement

All H atoms were fixed geometrically and treated as riding with C—H = 0.93–0.98 Å with U_iso_(H) = 1.2–1.5U_eq_(C). In the absence of significant anomalous scattering, the absolute configuration could not be reliably determined and thus 2403 Friedel pairs were merged.

### Crystal data

**Table:**

C_30_H_42_O_3_	*F*(000) = 492
*M**_r_* = 450.64	*D*_x_ = 1.118 Mg m^−^^3^
Monoclinic, *P*2_1_	Mo *K*α radiation, λ = 0.71073 Å
Hall symbol: P 2yb	Cell parameters from 15300 reflections
*a* = 6.6699 (2) Å	θ = 0.7–26.1°
*b* = 6.6335 (2) Å	µ = 0.07 mm^−^^1^
*c* = 30.2948 (8) Å	*T* = 298 K
β = 92.799 (1)°	Prism, colourless
*V* = 1338.79 (7) Å^3^	0.28 × 0.17 × 0.12 mm
*Z* = 2	

### Data collection

**Table:**

Bruker X8 APEX CCD area-detector diffractometer	2604 reflections with *I* > 2σ(*I*)
Radiation source: fine-focus sealed tube	*R*_int_ = 0.030
graphite	θ_max_ = 26.1°, θ_min_ = 0.7°
φ and ω scans	*h* = −8→8
15300 measured reflections	*k* = −8→8
2914 independent reflections	*l* = −37→37

### Refinement

**Table:**

Refinement on *F*^2^	Primary atom site location: structure-invariant direct methods
Least-squares matrix: full	Secondary atom site location: difference Fourier map
*R*[*F*^2^ > 2σ(*F*^2^)] = 0.043	Hydrogen site location: inferred from neighbouring sites
*wR*(*F*^2^) = 0.109	H-atom parameters constrained
*S* = 1.05	*w* = 1/[σ^2^(*F*_o_^2^) + (0.0425*P*)^2^ + 0.3687*P*] where *P* = (*F*_o_^2^ + 2*F*_c_^2^)/3
2914 reflections	(Δ/σ)_max_ < 0.001
304 parameters	Δρ_max_ = 0.17 e Å^−^^3^
1 restraint	Δρ_min_ = −0.12 e Å^−^^3^

### Fractional atomic coordinates and isotropic or equivalent isotropic displacement parameters (Å^2^)

**Table:**

	*x*	*y*	*z*	*U*_iso_*/*U*_eq_	
C1	−0.4278 (5)	0.3168 (6)	0.92476 (10)	0.0684 (9)	
H1	−0.5086	0.2029	0.9263	0.087 (11)*	
C2	−0.3866 (6)	0.4312 (7)	0.96587 (11)	0.0807 (12)	
H2A	−0.3027	0.3501	0.9860	0.097*	
H2B	−0.5123	0.4557	0.9798	0.097*	
C3	−0.2841 (5)	0.6299 (7)	0.95871 (9)	0.0705 (9)	
H3A	−0.2202	0.6748	0.9864	0.085*	
H3B	−0.3840	0.7295	0.9495	0.085*	
C4	−0.1256 (4)	0.6164 (5)	0.92373 (8)	0.0494 (6)	
C5	−0.0308 (5)	0.8240 (5)	0.91750 (9)	0.0623 (8)	
H5A	0.0449	0.8611	0.9444	0.075*	
H5B	−0.1366	0.9229	0.9125	0.075*	
C6	0.1087 (5)	0.8304 (5)	0.87881 (9)	0.0598 (8)	
H6A	0.1571	0.9670	0.8751	0.072*	
H6B	0.2238	0.7442	0.8853	0.072*	
C7	0.0003 (4)	0.7606 (4)	0.83602 (8)	0.0423 (6)	
H7	−0.1100	0.8554	0.8296	0.051*	
C8	−0.0936 (4)	0.5527 (4)	0.84179 (8)	0.0450 (6)	
H8A	0.0115	0.4537	0.8475	0.054*	
H8B	−0.1682	0.5146	0.8148	0.054*	
C9	−0.2345 (4)	0.5549 (4)	0.88021 (8)	0.0424 (6)	
H9	−0.3318	0.6624	0.8733	0.051*	
C10	−0.3577 (4)	0.3643 (5)	0.88525 (9)	0.0502 (7)	
C11	−0.4056 (6)	0.2386 (6)	0.84572 (12)	0.0796 (10)	
H11A	−0.4834	0.1240	0.8540	0.119*	
H11B	−0.2833	0.1934	0.8335	0.119*	
H11C	−0.4812	0.3168	0.8241	0.119*	
C12	0.0350 (5)	0.4630 (7)	0.93871 (11)	0.0737 (10)	
H12A	0.1411	0.4637	0.9184	0.111*	
H12B	−0.0236	0.3309	0.9394	0.111*	
H12C	0.0885	0.4980	0.9677	0.111*	
C13	0.1364 (4)	0.7703 (4)	0.79758 (9)	0.0449 (6)	
C14	0.2112 (6)	0.6149 (5)	0.77756 (12)	0.0845 (12)	
H14A	0.2901	0.6342	0.7534	0.101*	
H14B	0.1855	0.4851	0.7874	0.101*	
C15	0.1767 (4)	0.9763 (4)	0.78202 (8)	0.0426 (6)	
C16	0.3228 (3)	1.1350 (4)	0.72013 (8)	0.0415 (6)	
C17	0.5257 (3)	1.1794 (4)	0.70471 (8)	0.0436 (6)	
C18	0.6835 (4)	1.0992 (7)	0.72518 (11)	0.0753 (11)	
H18A	0.6681	1.0147	0.7493	0.090*	
H18B	0.8110	1.1266	0.7156	0.090*	
C19	0.5274 (3)	1.3227 (4)	0.66603 (7)	0.0417 (6)	
H19	0.4349	1.4330	0.6721	0.050*	
C20	0.4477 (4)	1.2199 (6)	0.62350 (8)	0.0591 (8)	
H20A	0.5305	1.1036	0.6176	0.071*	
H20B	0.3119	1.1728	0.6273	0.071*	
C21	0.4484 (4)	1.3633 (6)	0.58433 (8)	0.0630 (9)	
H21A	0.3514	1.4696	0.5886	0.076*	
H21B	0.4058	1.2903	0.5578	0.076*	
C22	0.6533 (4)	1.4587 (5)	0.57758 (8)	0.0508 (7)	
C23	0.7253 (4)	1.5623 (4)	0.62083 (8)	0.0415 (6)	
H23	0.6203	1.6600	0.6272	0.050*	
C24	0.7336 (4)	1.4171 (4)	0.65969 (8)	0.0447 (6)	
H24A	0.7777	1.4886	0.6864	0.054*	
H24B	0.8303	1.3115	0.6546	0.054*	
C25	0.9128 (4)	1.6862 (5)	0.61507 (9)	0.0529 (7)	
C26	0.9537 (5)	1.7582 (5)	0.57536 (11)	0.0681 (9)	
H26	1.0674	1.8384	0.5736	0.074 (10)*	
C27	0.8326 (6)	1.7205 (7)	0.53396 (11)	0.0801 (11)	
H27A	0.9085	1.6354	0.5148	0.096*	
H27B	0.8080	1.8477	0.5189	0.096*	
C28	0.6343 (5)	1.6208 (7)	0.54171 (10)	0.0717 (9)	
H28A	0.5824	1.5601	0.5144	0.086*	
H28B	0.5391	1.7223	0.5503	0.086*	
C29	1.0455 (5)	1.7295 (6)	0.65482 (12)	0.0789 (10)	
H29A	1.1575	1.8094	0.6465	0.118*	
H29B	1.0934	1.6050	0.6676	0.118*	
H29C	0.9712	1.8020	0.6761	0.118*	
C30	0.8016 (6)	1.2973 (6)	0.56362 (11)	0.0736 (10)	
H30A	0.9330	1.3554	0.5624	0.110*	
H30B	0.7596	1.2460	0.5350	0.110*	
H30C	0.8052	1.1893	0.5847	0.110*	
O1	0.3244 (3)	0.9824 (3)	0.75129 (6)	0.0519 (5)	
O2	0.0998 (3)	1.1250 (3)	0.79450 (7)	0.0590 (5)	
O3	0.1723 (3)	1.2153 (4)	0.70730 (6)	0.0600 (6)	

### Atomic displacement parameters (Å^2^)

**Table:**

	*U*^11^	*U*^22^	*U*^33^	*U*^12^	*U*^13^	*U*^23^
C1	0.0541 (16)	0.078 (2)	0.074 (2)	−0.0201 (17)	0.0141 (14)	0.0170 (19)
C2	0.077 (2)	0.105 (3)	0.0628 (19)	−0.015 (2)	0.0278 (16)	0.017 (2)
C3	0.084 (2)	0.084 (2)	0.0460 (15)	−0.007 (2)	0.0241 (15)	−0.0028 (18)
C4	0.0552 (15)	0.0556 (16)	0.0378 (12)	−0.0088 (14)	0.0081 (10)	0.0062 (13)
C5	0.084 (2)	0.061 (2)	0.0425 (14)	−0.0198 (18)	0.0090 (13)	−0.0091 (14)
C6	0.0707 (18)	0.0589 (19)	0.0502 (15)	−0.0273 (16)	0.0081 (12)	−0.0005 (15)
C7	0.0535 (14)	0.0331 (13)	0.0411 (12)	−0.0028 (11)	0.0101 (10)	0.0045 (11)
C8	0.0552 (14)	0.0405 (14)	0.0399 (12)	−0.0079 (12)	0.0086 (10)	0.0021 (11)
C9	0.0423 (12)	0.0421 (14)	0.0433 (13)	0.0021 (11)	0.0086 (10)	0.0071 (11)
C10	0.0407 (13)	0.0517 (17)	0.0583 (15)	−0.0056 (12)	0.0031 (11)	0.0072 (14)
C11	0.079 (2)	0.072 (2)	0.089 (2)	−0.035 (2)	0.0108 (17)	−0.003 (2)
C12	0.0582 (17)	0.095 (3)	0.0669 (18)	−0.0035 (18)	−0.0069 (14)	0.032 (2)
C13	0.0557 (14)	0.0345 (13)	0.0458 (13)	−0.0037 (11)	0.0147 (11)	0.0045 (11)
C14	0.128 (3)	0.0383 (17)	0.093 (2)	−0.002 (2)	0.067 (2)	0.0088 (19)
C15	0.0462 (13)	0.0386 (14)	0.0435 (13)	−0.0029 (12)	0.0084 (10)	0.0055 (12)
C16	0.0401 (13)	0.0424 (14)	0.0428 (12)	−0.0048 (12)	0.0091 (10)	0.0047 (12)
C17	0.0383 (12)	0.0520 (16)	0.0411 (12)	−0.0035 (11)	0.0082 (9)	0.0067 (12)
C18	0.0452 (15)	0.105 (3)	0.0771 (19)	0.0025 (18)	0.0132 (13)	0.042 (2)
C19	0.0386 (11)	0.0476 (16)	0.0396 (12)	−0.0052 (12)	0.0074 (9)	0.0040 (12)
C20	0.0589 (16)	0.072 (2)	0.0471 (14)	−0.0277 (16)	0.0050 (12)	0.0014 (16)
C21	0.0638 (17)	0.086 (2)	0.0390 (13)	−0.0177 (18)	−0.0034 (11)	0.0025 (16)
C22	0.0540 (14)	0.0620 (18)	0.0371 (12)	−0.0056 (14)	0.0090 (10)	0.0056 (14)
C23	0.0428 (12)	0.0409 (14)	0.0418 (12)	0.0014 (11)	0.0114 (10)	0.0023 (11)
C24	0.0423 (12)	0.0508 (17)	0.0411 (12)	−0.0091 (12)	0.0025 (9)	0.0049 (12)
C25	0.0507 (14)	0.0443 (16)	0.0647 (16)	−0.0053 (13)	0.0124 (12)	0.0059 (14)
C26	0.0652 (18)	0.061 (2)	0.079 (2)	−0.0125 (17)	0.0203 (15)	0.0178 (18)
C27	0.095 (2)	0.083 (3)	0.0640 (19)	−0.007 (2)	0.0216 (17)	0.032 (2)
C28	0.078 (2)	0.087 (3)	0.0501 (16)	−0.004 (2)	0.0049 (14)	0.0217 (18)
C29	0.070 (2)	0.075 (3)	0.091 (2)	−0.032 (2)	0.0022 (17)	0.008 (2)
C30	0.094 (2)	0.066 (2)	0.0630 (19)	0.001 (2)	0.0318 (17)	−0.0088 (18)
O1	0.0483 (9)	0.0472 (11)	0.0620 (10)	0.0037 (9)	0.0215 (8)	0.0176 (10)
O2	0.0766 (13)	0.0362 (10)	0.0664 (12)	−0.0011 (10)	0.0270 (10)	0.0048 (10)
O3	0.0373 (9)	0.0784 (15)	0.0652 (11)	−0.0008 (10)	0.0099 (8)	0.0263 (12)

### Geometric parameters (Å, °)

**Table:**

C1—C10	1.344 (4)	C16—O3	1.185 (3)
C1—C2	1.473 (5)	C16—O1	1.384 (3)
C1—H1	0.9300	C16—C17	1.482 (3)
C2—C3	1.506 (6)	C17—C18	1.308 (4)
C2—H2A	0.9700	C17—C19	1.509 (3)
C2—H2B	0.9700	C18—H18A	0.9300
C3—C4	1.535 (3)	C18—H18B	0.9300
C3—H3A	0.9700	C19—C20	1.530 (4)
C3—H3B	0.9700	C19—C24	1.532 (3)
C4—C9	1.529 (4)	C19—H19	0.9800
C4—C12	1.531 (5)	C20—C21	1.521 (4)
C4—C5	1.531 (4)	C20—H20A	0.9700
C5—C6	1.532 (4)	C20—H20B	0.9700
C5—H5A	0.9700	C21—C22	1.529 (4)
C5—H5B	0.9700	C21—H21A	0.9700
C6—C7	1.525 (4)	C21—H21B	0.9700
C6—H6A	0.9700	C22—C28	1.530 (4)
C6—H6B	0.9700	C22—C30	1.531 (4)
C7—C13	1.512 (3)	C22—C23	1.536 (4)
C7—C8	1.528 (4)	C23—C25	1.514 (4)
C7—H7	0.9800	C23—C24	1.520 (3)
C8—C9	1.531 (3)	C23—H23	0.9800
C8—H8A	0.9700	C24—H24A	0.9700
C8—H8B	0.9700	C24—H24B	0.9700
C9—C10	1.520 (4)	C25—C26	1.335 (4)
C9—H9	0.9800	C25—C29	1.487 (4)
C10—C11	1.481 (5)	C26—C27	1.479 (5)
C11—H11A	0.9600	C26—H26	0.9300
C11—H11B	0.9600	C27—C28	1.507 (5)
C11—H11C	0.9600	C27—H27A	0.9700
C12—H12A	0.9600	C27—H27B	0.9700
C12—H12B	0.9600	C28—H28A	0.9700
C12—H12C	0.9600	C28—H28B	0.9700
C13—C14	1.307 (4)	C29—H29A	0.9600
C13—C15	1.475 (4)	C29—H29B	0.9600
C14—H14A	0.9300	C29—H29C	0.9600
C14—H14B	0.9300	C30—H30A	0.9600
C15—O2	1.182 (3)	C30—H30B	0.9600
C15—O1	1.389 (3)	C30—H30C	0.9600
			
C10—C1—C2	125.0 (3)	O3—C16—C17	125.3 (2)
C10—C1—H1	117.5	O1—C16—C17	112.6 (2)
C2—C1—H1	117.5	C18—C17—C16	119.7 (2)
C1—C2—C3	113.4 (3)	C18—C17—C19	126.0 (2)
C1—C2—H2A	108.9	C16—C17—C19	114.3 (2)
C3—C2—H2A	108.9	C17—C18—H18A	120.0
C1—C2—H2B	108.9	C17—C18—H18B	120.0
C3—C2—H2B	108.9	H18A—C18—H18B	120.0
H2A—C2—H2B	107.7	C17—C19—C20	111.0 (2)
C2—C3—C4	112.4 (3)	C17—C19—C24	113.3 (2)
C2—C3—H3A	109.1	C20—C19—C24	110.71 (19)
C4—C3—H3A	109.1	C17—C19—H19	107.2
C2—C3—H3B	109.1	C20—C19—H19	107.2
C4—C3—H3B	109.1	C24—C19—H19	107.2
H3A—C3—H3B	107.9	C21—C20—C19	111.3 (3)
C9—C4—C12	112.0 (3)	C21—C20—H20A	109.4
C9—C4—C5	108.4 (2)	C19—C20—H20A	109.4
C12—C4—C5	110.3 (3)	C21—C20—H20B	109.4
C9—C4—C3	107.3 (2)	C19—C20—H20B	109.4
C12—C4—C3	109.2 (3)	H20A—C20—H20B	108.0
C5—C4—C3	109.6 (3)	C20—C21—C22	113.6 (2)
C4—C5—C6	112.9 (3)	C20—C21—H21A	108.9
C4—C5—H5A	109.0	C22—C21—H21A	108.9
C6—C5—H5A	109.0	C20—C21—H21B	108.9
C4—C5—H5B	109.0	C22—C21—H21B	108.9
C6—C5—H5B	109.0	H21A—C21—H21B	107.7
H5A—C5—H5B	107.8	C21—C22—C28	110.0 (2)
C7—C6—C5	111.2 (2)	C21—C22—C30	109.9 (3)
C7—C6—H6A	109.4	C28—C22—C30	109.1 (2)
C5—C6—H6A	109.4	C21—C22—C23	108.4 (2)
C7—C6—H6B	109.4	C28—C22—C23	107.7 (3)
C5—C6—H6B	109.4	C30—C22—C23	111.7 (2)
H6A—C6—H6B	108.0	C25—C23—C24	115.7 (2)
C13—C7—C6	111.4 (2)	C25—C23—C22	111.7 (2)
C13—C7—C8	113.0 (2)	C24—C23—C22	112.0 (2)
C6—C7—C8	110.9 (2)	C25—C23—H23	105.4
C13—C7—H7	107.1	C24—C23—H23	105.4
C6—C7—H7	107.1	C22—C23—H23	105.4
C8—C7—H7	107.1	C23—C24—C19	110.9 (2)
C7—C8—C9	110.4 (2)	C23—C24—H24A	109.5
C7—C8—H8A	109.6	C19—C24—H24A	109.5
C9—C8—H8A	109.6	C23—C24—H24B	109.5
C7—C8—H8B	109.6	C19—C24—H24B	109.5
C9—C8—H8B	109.6	H24A—C24—H24B	108.0
H8A—C8—H8B	108.1	C26—C25—C29	121.4 (3)
C10—C9—C4	111.8 (2)	C26—C25—C23	120.3 (3)
C10—C9—C8	115.1 (2)	C29—C25—C23	118.3 (2)
C4—C9—C8	112.0 (2)	C25—C26—C27	125.2 (3)
C10—C9—H9	105.7	C25—C26—H26	117.4
C4—C9—H9	105.7	C27—C26—H26	117.4
C8—C9—H9	105.7	C26—C27—C28	112.9 (3)
C1—C10—C11	121.2 (3)	C26—C27—H27A	109.0
C1—C10—C9	119.9 (3)	C28—C27—H27A	109.0
C11—C10—C9	118.9 (2)	C26—C27—H27B	109.0
C10—C11—H11A	109.5	C28—C27—H27B	109.0
C10—C11—H11B	109.5	H27A—C27—H27B	107.8
H11A—C11—H11B	109.5	C27—C28—C22	112.1 (3)
C10—C11—H11C	109.5	C27—C28—H28A	109.2
H11A—C11—H11C	109.5	C22—C28—H28A	109.2
H11B—C11—H11C	109.5	C27—C28—H28B	109.2
C4—C12—H12A	109.5	C22—C28—H28B	109.2
C4—C12—H12B	109.5	H28A—C28—H28B	107.9
H12A—C12—H12B	109.5	C25—C29—H29A	109.5
C4—C12—H12C	109.5	C25—C29—H29B	109.5
H12A—C12—H12C	109.5	H29A—C29—H29B	109.5
H12B—C12—H12C	109.5	C25—C29—H29C	109.5
C14—C13—C15	120.2 (2)	H29A—C29—H29C	109.5
C14—C13—C7	125.5 (3)	H29B—C29—H29C	109.5
C15—C13—C7	114.3 (2)	C22—C30—H30A	109.5
C13—C14—H14A	120.0	C22—C30—H30B	109.5
C13—C14—H14B	120.0	H30A—C30—H30B	109.5
H14A—C14—H14B	120.0	C22—C30—H30C	109.5
O2—C15—O1	121.6 (2)	H30A—C30—H30C	109.5
O2—C15—C13	125.6 (2)	H30B—C30—H30C	109.5
O1—C15—C13	112.8 (2)	C16—O1—C15	119.8 (2)
O3—C16—O1	122.1 (2)		

### Hydrogen-bond geometry (Å, °)

**Table:**

*D*—H···*A*	*D*—H	H···*A*	*D*···*A*	*D*—H···*A*
C14—H14B···O2^i^	0.93	2.47	3.378 (4)	166
C18—H18B···O3^ii^	0.93	2.51	3.419 (3)	168

Symmetry codes: (i) *x*, *y*−1, *z*; (ii) *x*+1, *y*, *z*.
